# Complexities and subtleties in the measurement and reporting of breastfeeding practices

**DOI:** 10.1186/1746-4358-6-5

**Published:** 2011-05-17

**Authors:** Debra J Hector

**Affiliations:** 1 Prevention Research Collaboration, University of Sydney, NSW 2006, Australia

## Abstract

**Background:**

Monitoring of breastfeeding is vital. However, infant feeding practices are difficult to assess at the population level. Although significant efforts have been made towards the consistent measurement and reporting of breastfeeding, few countries have successfully implemented a system to do so. Many inaccuracies, inconsistencies and issues remain. This paper highlights the main issues relating to the methods and indicators used to monitor breastfeeding, particularly exclusive breastfeeding, at the population level. In doing so, it aims to support progress in this area.

**Discussion:**

Indicators are used primarily for comparative purposes and should be broadly consistent with recommended practice; regarding exclusive breastfeeding this is 'to six months'. There are limitations to both main methods used to measure and report on breastfeeding: current status (often 24-hour recall), and longer-term recall. Issues relate to how age is considered within the analysis and interpretation of data, including boundary points or cut offs, as well as how breastfeeding practices are reported against different ages, especially regarding whether to use the preposition 'to' or 'at'. Other issues include the conversion from weeks to months, as well as the 'regular' *versus *'first' introduction of something other than breast milk, to signify the deviation from exclusive breastfeeding. Differences in how data are collected, and uncertainties around how data are interpreted, have led to the mixed and often inaccurate reporting of breastfeeding practices, particularly exclusive breastfeeding. Assuming a particular definition of exclusive breastfeeding, such as that of the World Health Organization, the period over which exclusive breastfeeding is measured and how it is determined in the survey are important in relation to indicator phrasing. Often compromises are made in data collected to report against exclusive breastfeeding, despite subsequent reporting of exclusivity.

**Summary:**

Indicators to report on breastfeeding must be carefully phrased. The commonly reported indicator *exclusive breastfeeding at six months *is redundant and should never be reported, while the more appropriate indicator *exclusive breastfeeding to six months *may not be sufficiently sensitive to change, and cannot be measured by current status methods alone. Importantly, indicators must accurately reflect the data collected to ensure valid comparisons between surveys.

## Background

It is beyond conjecture that breastfeeding confers multiple short-term and long-term health benefits to infants and mothers, with many studies showing a dose-response relationship. Also, particular health benefits are linked to exclusive breastfeeding for the first months of life, probably to six months. The established health benefits of breastfeeding, or conversely the risks of infant formula or artificial milk feeding, render breastfeeding an important area for population health. Accordingly, as per the truism 'we measure what we value and value what we measure', we must measure and report on breastfeeding.

Significant efforts have been made nationally and internationally to support the consistent, standardised collection of data and reporting of breastfeeding practices. For example, the document *Towards a national system for monitoring breastfeeding in Australia: recommendation for population indicators, definitions and next steps *[1] was aimed at standardising the collection and reporting of breastfeeding data in Australia. The World Health Organization (WHO) had earlier, in 1991, published a set of recommended indicators [2] that were recently revised [3], which aimed to standardise the measurement and reporting of breastfeeding practices worldwide. Other countries have striven to report on breastfeeding in a consistent manner [4,5]. Despite these efforts, few countries have successfully implemented a whole-of-country system for measuring and reporting breastfeeding practices. In the United States (US) for example, Chapman and Pérez-Escamilla concluded that "*obviously further research and expert committee consultation are needed to make progress towards this goal*" [5] (p. 148).

Many inaccuracies and inconsistencies in the monitoring and reporting of breastfeeding remain. This article provides some perspectives on this complex area, highlighting and summarising the major issues involved, particularly with regard to exclusive breastfeeding, as well as noting some discrepancies in the recent literature. It aims to support progress towards consistency in the measurement and reporting of breastfeeding, within Australia and internationally.

## Discussion

### What is an indicator?

Simple, valid and reliable indicators are crucial to track progress and guide investment. An indicator is "used in the field of public health monitoring and surveillance to describe a specific and measurable statistical construct for monitoring progress towards a goal (a broad statement of desired improvement)" [1] (p. xii). Indictors are therefore primarily for comparative purposes. To draw conclusions over a period of time, or across surveys, decision-makers must be certain they are looking at data which measure the same phenomenon. The definition of an indicator must therefore remain consistent each time it is measured and reported. Additionally, care must be taken to use the same measurement instrument or data collection method to ensure consistent data are collected. Interpretation of the data must be consistent too. Consequently, the phrasing of an indicator, and how the data are used to report against the indicator, are paramount.

### Infant feeding guidelines

Indicators must be broadly consistent with standards, recommendations and best-practice. Although most allergists and immunologists in Australia recommend the introduction of solid foods between four and six months and not beyond six months [6,7], the recommended duration of exclusive breastfeeding is six months [8,9]. The current National Health and Medical Research Council (NHMRC) Infant Feeding Guidelines in Australia related to breastfeeding are 'exclusive breastfeeding for the first six months' and to 'continue breastfeeding for at least 12 months' [9]. Also, current NHMRC guidelines indicate that breastfeeding should be complemented with appropriate, hygienically prepared food from *around *six months of age. This 'looseness' in the wording for the timing of introduction of solid foods caters for some infants being developmentally ready for solids prior to six months. However this creates some problems as it is juxtaposed with the recommendation for exclusive breastfeeding to six months.

### Measurement of breastfeeding

In Australia [1] the current recommendation for determining rates of exclusive breastfeeding, hence the introduction of substances other than breast milk, is by current status methods, often 24-hour recall, as recommended by the World Health Organization (WHO) [3]. The advantages of the 24-hour recall method are that it requires a small number of questions in order to report against many indicators important for policy and practice, the analysis and interpretation of the data is straightforward, and recall error is significantly reduced. Consequently, the method can be used to gain reliable estimates of trends in breastfeeding practices. In Australia it has been used in state-level surveys in New South Wales (NSW) and Queensland (QLD) - together with longer-term recall questions - and it has been used for data collection at the area health service level (South Eastern Sydney Illawarra Health), and state-wide in the Personal Health Record in NSW.

The other main method to measure and report on breastfeeding practices involves questions relating to prior practices involving longer-term (longer than seven days) recall, often since birth, in cross-sectional population surveys or generally over several months in cohort studies.

### Issues with current status methods

#### Misclassification of exclusive breastfeeding

The major criticism of the 24-hour recall method is that it misclassifies too many mothers as exclusively breastfeeding [10]; a proportion of mothers may be providing substances other than breast milk on an irregular, not daily, basis. Many research studies [11-15] and the Queensland Infant Nutrition Project [16] have shown that a large proportion of infants that were exclusively breastfed in the previous 24 hours were (a) not exclusively breastfed during the previous seven days, and/or, (b) not exclusively breastfed since birth.

To reduce these misclassification errors, one approach would be to ask the WHO recommended 24-hour recall question as a 'first pass' and then to ask 'follow-on' questions. Indeed, the WHO itself recommended ascertaining if the previous 24 hours was representative of usual practice. Questions could then be asked of the same respondents to determine whether the infant has been exclusively breastfed (a) in the previous seven days, and/or (b) since birth. There have been a number of suggestions as to what these questions might be, including 'Has the baby ever been given water, other fluids, or solids *since he or she was born*?', and if yes, followed by the question 'Was it given *regularly*?'[11]; and asking mothers to recall how old their infants were when they first fed other foods *at least 1 day per week *[13]. Other suggestions are 'Has any solid or liquid other than breast milk ever been given *since birth*, and if so, when was that done for the *first time*?' [14]; and, determining the 'occasional' as opposed to 'regular' use of breast milk substitutes, or even further describing frequency of solid foods intake [15]. These questions would need to be asked of all respondents, not just those that indicated that the infant was fed breast milk in the previous 24 hours. How these follow-on data would then be interpreted and used in relation to the current status data is not clear, however.

#### Sample size

Another issue with the 24-hour recall method is the need for large sample sizes of infants aged less than six months (0-5.99 months), an issue that would be compounded by using it as a 'first pass'. Most cross-sectional population level surveys in Australia have not had sufficient numbers of young infants to determine rates of exclusive breastfeeding using 24-hour recall. For example, among the 3252 children less than four years in the 1995 National Health Survey in Australia, only 358 were less than six months old, i.e. sample sizes for some months were smaller than 50 [17]. It has been determined that "*an increase in 5% of the rate of full breastfeeding between sample years, for example, could only reliably be detected from a sample size of about 1200 infants at six months of age (with a power of 80% and significance level of 5%)*" [1] (p. 41). Presumably the 'at six months' refers to infants aged 0 to less than six months.

#### Choice of indicator

Another potential inadequacy of the 24-hour recall method alone is that any indicator reported using this method can only report rates *at *each month of age, i.e. it is not possible to report against a single, definitive indicator relating to recommended practice such as 'exclusive breastfeeding *to *six months'. The WHO, cautioning that the indicator should not be interpreted as 'proportion of infants exclusively breastfed until six months of age', suggested that the indicator be 'exclusive breastfeeding *under *six months' [3] (p. 5-6). That is, they recommend averaging the rate of exclusive breastfeeding across infants aged 0 to less than six months of age (0-5.99 months). They considered that this indicator would be more sensitive to capturing changes over time. However, unless large numbers of infants aged each month of age from 0 to six months are quota sampled with similar numbers of infants at each month of age, then the percentage will not be comparable across surveys and countries. The WHO acknowledged that disaggregating the indicator is probably necessary [3] (p. 6). Agampodi et al warn against use of this indicator, noting that Sri Lankan health officials were reporting that 75.5% of infants were being exclusively breastfed to six months, when in fact the percentage at four to five months was 53.4% and rates of exclusive breastfeeding from birth to six completed months was likely to be as low as 20% [18].

### Issues with longer-term recall

The longer-term recall method commonly employs survival analysis techniques to determine duration and intensity of breastfeeding across the population. There are a number of limitations or issues associated with this method. First, there are problems of maternal recall. Second, there are issues with determining boundary points or cut-offs and use of prepositions. Third, questions can relate to the *first *or *regular *giving of something other than breast milk to determine cessation of exclusive breastfeeding, an issue also observed in the suggested 'second pass' questions associated with 24-hour recall indicated above. Fourth, there is no standard method of converting from weeks to months.

#### Maternal recall

The issue of maternal recall has been discussed extensively [1,10-16,19]. This problem can be overcome by use of a cohort, prospective study design to reduce the period of recall, although this is often deemed not practical, affordable, and/or necessary at the population level [1,14].

#### Boundary points, cut-offs and prepositions

The issue of boundary points has been less discussed. While the current status method clearly enables the reporting of breastfeeding practices at a particular age, in surveys containing questions related to longer-term recalled practice, e.g. since birth, the cut-offs or boundary points become less clear.

Survival analysis techniques including the Life-table and Kaplan-Meier methods have been used to determine breastfeeding rates in Australia and internationally and both techniques are equally applicable in most instances. Garden et al indicate that the Life Table method was adopted in their analyses because the duration of breastfeeding and age of the child are recorded as an interval (in months) rather than an exact date [20].

In survival analysis, prevalence rates of breastfeeding are usually sourced from survey questions asking age at which breastfeeding stopped, and an infant is assumed to have been breastfed for all of the time up until the age of cessation, although it is not clear if they are also considered to be breastfed at the month of cessation. For example, "*it is assumed that someone who stopped breastfeeding at a particular age was breastfeeding for all the time up until the age of cessation (for example a child who stopped being breastfed at 4 months is assumed to have been breastfed at ages 1, 2 and 3 months)" *[1,20] (p. 54; p. 29). Presumably the infant is assumed to also have been breastfed at less than one month.

Exclusive breastfeeding ceases once something other than breast milk is introduced into the infant diet. The implication of this is that if a mother reports that she introduced solids to her infant when he/she was six months old, and had given the infant nothing other than breast milk until that time, then the infant was assumed to have been exclusively breastfed AT each month of age TO 6 months, i.e. AT < 1 month, AT 1 month, AT 2 months, AT 3 months, AT 4 months, and AT 5 months, but *not *AT 6 months. Similarly, if solid foods were introduced when the infant was five months old, then the infant was exclusively breastfed FOR 5 months, TO 5 months and AT each month of age UP TO 5 months but *not *AT 5 months.

Uncertainty remains however, where the boundary points for various breastfeeding practices are being set within the survival analyses, and/or if this tallies with the actual breastfeeding practice. How the raw data are 'used' within the survival analysis, and how the survival curves are interpreted - specifically how percentages are 'read off' from the curves, particularly from the different survival analysis methods - is often not evident. For example, are mothers who stop breastfeeding during the infant's first month included in the 'at less than one month' percentage, are they 'ignored', or are they included in the 'at one month' percentage?

The issue of 'boundary points' was raised in the UK Infant Feeding Survey 2005: "*when deriving certain parameters such as time of cessation of exclusive breastfeeding, then the boundary point needs to be defined. For example, if a mother responded that they first introduced formula at six weeks, should they be counted as being exclusive or not exclusive at six weeks? It was decided that in such a situation the baby would be counted as being exclusively breastfed UP TO six weeks, but not at the six week point itself. This same principle was applied for all different ages*." [21] (p. 41). However, despite raising this issue, the indicator was then reported as 'the proportion of all babies who are being exclusively breastfed AT specific ages' (also page 41). Hence the interpretation must be that the infant in their given example was not included in the rate of exclusive breastfeeding at six weeks. However, in accordance with the uncertain interpretation of output from survival analysis, it is not clear whether this is, in fact, the case.

The uncertainty around how data are analysed and interpreted has led to the mixed reporting of breastfeeding practices, exclusive breastfeeding in particular. Often the two prepositions, 'at' and 'to' (as well as 'for' and 'until', largely equivalent to 'to') are used interchangeably when describing rates of exclusive breastfeeding. Most commonly, the indicator 'exclusive breastfeeding AT six months' is used to describe data which may or may not accurately portray exclusive breastfeeding TO six months. 'Exclusive breastfeeding AT six months' is a redundant indicator, as solid foods are recommended to be introduced at six months of age. Not surprisingly therefore, many studies have determined exclusive breastfeeding at six months of age to be non-existent. For example, in the UK Infant Feeding Survey 2005 [21] (p. 23) exclusive breastfeeding was reported as: "*at six months the prevalence of exclusive breastfeeding was negligible in all [UK] countries (<1%)*". Other examples include "*at six months, none of the women was exclusively breastfeeding" *[22] and "*rates of exclusive breastfeeding amongst infants aged six months or younger were 39%" *[23]. Where reported exclusive breastfeeding rates at six months are high, e.g. "*exclusive breastfeeding rates at six months after birth (14.4%) were lower than desirable*" [24] (p. 122), it seems likely that these data more accurately reflect exclusive breastfeeding TO six months. Such data could also be reported as 'at five months', if the survey and analysis method permits.

#### 'First' or 'regular' introduction

As in the suggested options for follow-on questions when using 24-hour recall methods (see above), questions for longer-term recall differ in whether they ask about the *first *or *regular *introduction of substances other than breast milk. All of the population-level studies in the United States have used the *first *introduction of something other than breast milk [5]. In Australia research studies have often used *first *[25], whereas state-level and national-level surveys have tended to use *regular *or *first regular*. In NSW and QLD *regular *has been defined as 'once per day' and in national surveys *regular *has been left open to respondent interpretation. No studies have compared the response differences, if any, to these two types of questions.

It is well-established that infants who receive infant formula in hospital have much poorer breastfeeding outcomes [26-28]; much less is known about the clinical relevance of small amounts of formula supplementation in hospital [29] or later in the infant period among otherwise exclusively breastfed babies. There are known effects of formula-feeding on the digestive tract [10,30], but the impact of these effects on health outcomes has not been specifically examined. The problems of giving water to newborns have been summarised [31].

Infants may be given formula in hospital and then breastfed exclusively post-discharge [32]. In the United States, rates of exclusive breastfeeding for the first few days of life (up to four days) were about 20% lower when measured through maternity database collections compared to longer-term recall of 19-35 months through the National Immunization Survey [29]. These data suggest that many infants were given formula in hospital, but this isn't remembered or known by mothers via longer-term recall. Another possibility is that mothers might consider small amounts of early formula as not important and therefore not report it [29]. Flaherman et al summarise the literature regarding maternal recall of infant feeding practices and conclude that there is no available literature supporting the accurate recall of early formula use beyond six months postpartum [29]. They suggest that an appropriate survey question could be '*how much formula did your child receive (if any) during newborn hospitalization?*'.

Accurate data on the 'proportion of infants exclusively breastfed since birth', in the strictest, exact sense, are important for research relating breastfeeding practices to specific health outcomes, particularly in relation to mother-to-child-transmission of Human Immunodeficiency Virus [11,12,15]. Overestimation of rates of exclusive breastfeeding due to not precisely measuring it in its strictest, exact sense, since birth, leads to an underestimation of the health benefits of this breastfeeding practice. However, whether it is important to measure exclusive breastfeeding in its exact sense, since birth, in monitoring surveys at the population level is arguable.

#### Conversion from weeks to months

Infant feeding recommendations are indicated in months and most often breastfeeding practices are reported in months, yet many data collections allow mothers to respond in days and/or weeks, particularly during the early months, with some studies only recording breastfeeding in weeks. There is no standard way of converting between weeks and months. Often the number of weeks in a year is divided by the number of months so that 1 month is assumed equal to 4.33 weeks, 2 months assumed equal to 8.66 weeks, etc. In other instances 8 weeks is considered to be equal to 2 months and 24 weeks is considered to be equal to 6 months, for example. In a study by Scott et al responses were allowed only in weeks [33]; they observed a peak at 16 weeks around the introduction of solid foods, which suggests that perhaps mothers interpret 16 weeks as 4 months. How mothers consider infant age when they report on infant feeding practices has not been explored.

### Definitions and other wording issues

Calls for consistency in breastfeeding definitions began in the late 1980s [34] and have occurred more recently [10,35]. However, I contend that the main issue now is not one of definition of breastfeeding practices, at least for exclusive breastfeeding. The WHO definition of exclusive breastfeeding is generally accepted as a reasonable description of what is allowed to be received by an infant, other than breast milk, i.e. drops or syrups consisting of vitamins, mineral supplements or medicines [2,3], and can be successfully applied to all survey methods and research studies. Rather, there are two issues that emerge when considering the reporting of exclusive breastfeeding: (i) the period over which exclusive breastfeeding is determined, and (ii) the text description of how exclusive breastfeeding is determined.

The period over which exclusive breastfeeding is recalled and measured varies between survey methods. In the current status method it is usually 24 hours, 48 hours or 7-days, whereas in longer-term recall or prospective studies it is usually 'since birth'; the latter regardless of whether *first *or *regular *introduction signifies the deviation from exclusivity. Therefore an indicator using current status or short-term recall methods might be more accurately described in a more cumbersome manner involving the time period of recall. This was recommended by Binns et al, who suggested that data collected using the 24-hour recall method be referred to as the '*24-h full breastfeeding rate at "X" months*' [10] (p. 179). However, reporting those exclusively breastfed in the previous 24 hours as a fully breastfed rate only takes account of potential misclassification due to water or watery drinks on other days - infant formula and/or solid foods can also be given intermittently. Exclusive breastfeeding, as opposed to full breastfeeding, according to the WHO definitions, is easily measureable and reportable using the 24-hour recall method, hence the indicator in this instance would most appropriately be the '*24-h exclusive breastfeeding rate at "X" months*'.

Often the WHO definition is accepted as 'true', but then compromises are made in terms of the survey questions, disabling accurate reporting against the definition despite the indicator 'exclusive breastfeeding' being reported. For example, Lande et al state in the body text of their paper that: "*Exclusive breastfeeding at 0.5-5.5 mo included infants given water (use of water was not covered by the retrospective questions). Breast milk was their only energy source and therefore we preferred to use the category exclusive breastfeeding rather than predominant breastfeeding*." [36] (p. 154). The inclusion or exclusion of the giving of water to infants in the monitoring questions used to report against exclusive breastfeeding has been recently commented on in studies in the US [5,29]. Similarly, as indicated by Binns et al [10], the NSW Population Health Survey reports exclusive breastfeeding as the indicator, but in the body text describes the indicator as only taking into account water and fruit juice, not other watery drinks [20,37]; as well as allowing the infant to receive substances other than breast milk irregularly. In comparison, Kristiansen et al, for example, clearly indicate that infants were classified as exclusively breastfed at a particular age if they had received only breast milk and had not been introduced to any additional food or drink, not even water, but could have received vitamin-mineral supplements [38].

Perhaps what is needed is a 'prohibition' on the reporting of exclusive breastfeeding unless the data collection methods allow the accurate reporting against the WHO definition or the indicator stipulates the deviation from the WHO definition, as well as including the time period over which it is measured.

Another 'wording' issue relates to bottle feeding. In 2008 the WHO revised their optional indicators to include bottle feeding as the '*proportion of children 0-23.9 months of age who are fed with a bottle*' (during the previous day) [3]. This indicator includes the giving of expressed breast milk in a bottle and was in recognition of the potential interference of bottle feeding with optimal breastfeeding practices, as well as the association with increased incidence of diarrhoeal disease. Thulier comments on the potential differential effect of bottle feeding *versus *breast feeding of breast milk on some health outcomes, notably obesity; however despite purporting to, her proposed definitions and resultant groups for infant feeding do not specifically incorporate this distinction [35]. Indeed, Thulier's suggested alternative definitions for infant breast feeding offer considerably less consistency than the WHO definitions and only relate to a largely unmeasurable intensity of breastfeeding, particularly in relation to population monitoring, but probably even for the stated purpose of research.

Finally, although of less importance than some of the other issues raised, there is a lack of consistency around survey questions and indicators in relation to whether they are phrased as a maternal behaviour or as an infant behaviour [5]. Different terms include breastfed (breast fed, breast-fed), breast milk (breastmilk) fed, expressed breast milk fed, breastfeeding (breast-feeding, breast feeding), breast milk feeding, and expressed breast milk feeding.

## Summary

The subtleties and complexities discussed in this paper in regard to monitoring breastfeeding, for example around choice of boundary points and prepositions, the issue of first or regular introduction of substances other than breast milk to indicate cessation of exclusive breastfeeding, the period over which exclusivity is measured as well as compromises in survey questions and therefore data collected, require that breastfeeding indicators be carefully phrased. Consistency is required in converting survey responses from weeks to months, and in how output from survival analyses is interpreted.

Particular care should be taken around the use of the prepositions 'at' or 'to' in relation to the measured breastfeeding practice. The indicator *exclusive breastfeeding at six months *should never be reported. The more appropriate single indicator *exclusive breastfeeding to six months*, i.e. the introduction of something other than breast milk at (or after) six months among otherwise exclusively breastfed infants, relates to recommended practice. However this indicator cannot be reported against using current status methods alone. Furthermore, the sensitivity of this indicator is questioned as, even with substantial promotion efforts, this indicator is unlikely to vary much over time, particularly with the prevailing advice from allergists and immunologists to introduce solid foods between four to six months. The loose NHMRC recommendation around the introduction of solid foods further complicates the matter.

If the phrasing of breastfeeding indicators is determined independently of survey and analytical methods, then these methods must in some way accompany the indicator each and every time it is communicated, to avoid misinterpretation or erroneous comparisons between surveys. Optimally, the survey questions, methods of analysis, interpretation of analysed data, and subsequent reporting, need to be considered in tandem with indicator phrasing, not in isolation. A set of indicators chosen must adequately encompass all of the issues. Ultimately, a reported indicator must accurately describe the data collected.

## Competing interests

The perspective in this paper is the opinion and work of the author only. The author was the lead consultant on a Commonwealth-funded project (Commonwealth Department of Health & Ageing) to review and update breastfeeding indicators for use in Australia, in 2008. She also participated in a national workshop to reach consensus on breastfeeding indicators to be used in Australia in relation to the National Breastfeeding Strategy, in December 2010; after drafting this manuscript.
